# Changes in Blood Biomarkers of Angiogenesis and Immune Modulation after Radiation Therapy and Their Association with Outcomes in Thoracic Malignancies

**DOI:** 10.3390/cancers13225725

**Published:** 2021-11-16

**Authors:** Eleni Gkika, Sonja Adebahr, Anton Brenner, Tanja Schimek-Jasch, Gianluca Radicioni, Jan-Philipp Exner, Alexander Rühle, Simon K. B. Spohn, Ilinca Popp, Constantinos Zamboglou, Tanja Sprave, Elke Firat, Gabriele Niedermann, Nils Henrik Nicolay, Ursula Nestle, Anca-Ligia Grosu, Dan G. Duda

**Affiliations:** 1 University Medical Center Freiburg, Department of Radiation Oncology, Faculty of Medicine, University of Freiburg, 79106 Freiburg, Germany; sonja.adebahr@uniklinik-freiburg.de (S.A.); anton.brenner@uniklinik-freiburg.de (A.B.); tanja.schimek-jasch@uniklinik-freiburg.de (T.S.-J.); gianluca.radicioni@uniklinik-freiburg.de (G.R.); jan.exner@uniklinik-freiburg.de (J.-P.E.); alexander.ruehle@uniklinik-freiburg.de (A.R.); simon.spohn@uniklinik-freiburg.de (S.K.B.S.); ilinca.popp@uniklinik-freiburg.de (I.P.); constantinos.zamboglou@uniklinik-freiburg.de (C.Z.); tanja.sprave@uniklinik-freiburg.de (T.S.); elke.firat@uniklinik-freiburg.de (E.F.); Gabriele.niedermann@uniklinik-freiburg.de (G.N.); nils.nicolay@uniklinik-freiburg.de (N.H.N.); ursula.nestle@uniklinik-freiburg.de (U.N.); anca.grosu@uniklinik-freiburg.de (A.-L.G.); 2 German Cancer Consortium (DKTK), 79106 Freiburg, Germany; 3 German Cancer Research Center (DKFZ), 69120 Heidelberg, Germany; 4 Department of Radiation Oncology, Kliniken Maria Hilf, 41063 Moenchengladbach, Germany; 5 E. L. Steele Laboratories for Tumor Biology, Department of Radiation Oncology, Massachusetts General Hospital and Harvard Medical School, Boston, MA 02114, USA; duda@steele.mgh.harvard.edu

**Keywords:** radiotherapy, immune modulation, lung cancer, esophageal cancer

## Abstract

**Simple Summary:**

Radiation therapy can promote chemotaxis of cytotoxic T-lymphocytes by triggering the release of chemokines and altering the tumor’s vascular endothelium, triggering both pro- and anti-inflammatory immune responses and altering the tumor microenvironment. These effects of local irradiation may have systemic consequences and can be enhanced through the combination of available immune checkpoint blockers (ICBs). The study and validation of minimally invasive blood biomarkers for response and toxicity assessment are critical to stratify patients that would benefit from combination treatments. This exploratory prospective study evaluated the impact of thoracic radiotherapy approaches on the immune system using longitudinal assessment of a panel of blood biomarkers of angiogenesis and inflammation. We show that changes in circulating TNF-α, IL-6 and IL-8 levels could potentially indicate an early reduction in immunosuppression after radiotherapy. If validated in larger studies, these biomarker candidates might potentially help in optimally scheduling radiotherapy in combination with ICBs.

**Abstract:**

The effects of radiotherapy on systemic immunity remain to be fully characterized in a disease-specific manner. The aim of the study was to examine potential biomarkers of systemic immunomodulation when using radiotherapy for thoracic malignancies. Serial blood samples were collected from 56 patients with thoracic malignancies prior (RTbaseline), during (RTduring) and at the end of radiotherapy (RTend), as well as at the first (FU1) and second follow-up (FU2). The changes in serum levels of IL-10, IFN-γ, IL-12p70, IL-13, IL-1β, IL-4, IL-6, IL-8, TNF-α, bFGF, sFlt-1, PlGF, VEGF, VEGF-C, VEGF-D and HGF were measured by multiplexed array and tested for associations with clinical outcomes. We observed an increase in the levels of IL-10, IFN-γ, PlGF and VEGF-D and a decrease in those of IL-8, VEGF, VEGF-C and sFlt-1 during and at the end of radiotherapy. Furthermore, baseline concentration of TNF-α significantly correlated with OS. IL-6 level at RTend and FU1,2 correlated with OS (RTend: *p* = 0.039, HR: 1.041, 95% CI: 1.002–1.082, FU1: *p* = 0.001, HR: 1.139, 95% CI: 1.056–1.228, FU2: *p* = 0.017, HR: 1.101 95% CI: 1.018–1.192), while IL-8 level correlated with OS at RTduring and RTend (RTduring: *p* = 0.017, HR: 1.014, 95% CI: 1.002–1.026, RTend: *p* = 0.004, HR: 1.007, 95% CI: 1.061–1.686). In conclusion, serum levels of TNF-α, IL-6 and IL-8 are potential biomarkers of response to radiotherapy. Given the recent implementation of immunotherapy in lung and esophageal cancer, these putative blood biomarkers should be further validated and evaluated in the combination or sequential therapy setting.

## 1. Introduction

Radiotherapy is a mainstay of cancer treatment. Recent studies have demonstrated that in addition to cancer cell cytotoxicity, ionizing radiation could also have favorable immune-modulatory effects that trigger antitumor immune responses [1,2,3,4,5,6]. The potentiation of antitumor immune responses may be mediated by immunogenic cell death of cancer cells but also by changes in the tumor immune microenvironment and antigen presentation on the irradiated cancer cells [7,8]. Antigen-presenting cells migrate to the lymph nodes where they facilitate the priming of tumor-specific cytotoxic T-lymphocytes (CTLs) [9]. Tumor infiltration and activation of antigen-presenting cells and immune effector cells is mediated by several cytokines such as TNFα, IL-1β, IL-6 secreted in the tumor [10,11,12]. Radiotherapy alone can elicit antitumor T-cells that infiltrate the tumor and produce interferon-gamma (IFN-γ), which, in turn, induces PD-L1 expression on tumor cells [13]. Similarly, PD-L1 upregulation was driven by effector T-cell infiltration in a poorly immunogenic tumor after radiotherapy plus TGF-β blockade [14].

In several thoracic malignancies such as non-small-cell lung cancer (NSCLC), the combination of chemoradiation with immunotherapies in several settings (metastatic to curative treatment concepts) [15,16] has shown promise, and similar results have been also reported for esophageal cancers [17] in the adjuvant setting. These prior findings suggest that radiotherapy has both local and systemic effects on inflammation and antitumor immune responses. Thus, there is a rationale for combining checkpoint inhibitors (ICBs) with radiotherapy, as the radiation-induced immune activation of CTLs can be further boosted by ICBs or other immunomodulatory agents. The study and validation of minimally invasive blood biomarkers for response and toxicity assessment are critical to better understand and stratify patients with thoracic cancers that would benefit from combination treatments with radiotherapy.

## 2. Materials and Methods

### 2.1. Study Design

This trial was performed in accordance with the Declaration of Helsinki. Consecutive patients with histologically proven thoracic malignancies, including lung or esophageal cancer, thymoma or lung metastasis treated with thoracic radiotherapy, either as concurrent chemoradiation, postoperative radiotherapy, stereotactic body radiotherapy (SBRT) or palliative hypofractionated radiotherapy, participated over a period of 6 months. The study is an expansion of a prior study designed to evaluate the role of CCL18 in predicting radiation-induced lung disease [18]. After amending the protocol and receiving approval from the University of Freiburg Medical Center Ethics Committee, additional cytokine analyses were included according to a predefined research plan. The aim was to evaluate the impact of thoracic radiotherapy approaches on the immune system using longitudinal assessment of a panel of blood biomarkers of angiogenesis and inflammation. Patients received whole-body ^18^FDG PET/CTs or CTs as part of the initial staging procedure, which was assessed according to the UICC 7th Edition. Pulmonary function tests were routinely performed before treatment per standard of care.

### 2.2. Treatment

Radiotherapy planning was performed using 3D or 4D computed tomography (CT). Patients received thoracic radiotherapy using either normo-fractionated regimens (1.8–2 Gy per fraction) with curative intent up to a total dose of 54–66 Gy; hypofractionated regimens (3 Gy per fraction) for palliation up to a total dose of 30–36 Gy; or stereotactic body radiotherapy (SBRT) regimens using 7–12.5 Gy in 3–5 fractions prescribed at the 60% isodose every other day) as previously described [19]. Chemotherapy consisted of a platinum agent (carboplatin or cisplatin) either as monotherapy or in combination with vinorelbine for concurrent chemoradiation.

### 2.3. Response Evaluation and Toxicity

Endpoints assessed included treatment response and radiation-induced lung toxicity. These were evaluated weekly during radiotherapy, within 6–8 weeks after the end of radiotherapy, afterwards every 3 months during the 1st year, then every six months up to the 5th year and thereafter annually. Acute toxicity (up to 90 days after start of radiotherapy) was prospectively scored according to Common Terminology Criteria (CTC) version 4 and late toxicity according to the Radiation Therapy Oncology Group/European Organisation for Research and Treatment of Cancer (RTOG/EORTC) scoring system. Response was defined according to the Response Evaluation Criteria in Solid Tumors (RECIST) by CT and/or ^18^F-FDG PET/CT in case of suspected locoregional or distant disease progression.

### 2.4. Blood Biomarkers

Blood samples were collected prior to radiotherapy (RTbaseline), during (RTduring) at the end of treatment (RTend) and during the first (FU1) and second follow-up (FU2) (Supplementary Figure S1) Venous blood samples were obtained by venipuncture and processed according to standard operating procedures using Monovette serum tubes for serum processing. The serum levels of IL-10, IFN-γ, IL-12p70, IL-13, IL-1β, IL-4, IL-6, IL-8, TNF-α, bFGF, sFLT-1, PlGF, VEGF, VEGF-C and VEGF-D (Supplementary Table S1) were measured by multiplex array (MesoScale Discovery) and hepatocyte growth factor (HGF) by enzyme-linked immunosorbent assays (ELISA) (R&D Systems, Inc., Minneapolis, MN, USA) in the CLIA-certified facility at MGH Boston, USA, as previously described [20,21]. All measurements were performed in duplicate.

### 2.5. Statistical Analysis

The impact of different blood biomarker levels on overall survival (OS) was estimated using a Cox regression analysis. Furthermore, the up- or downregulation of the biomarkers as categorical variables was also correlated with OS using Cox regression. The observations for OS were censored at the date of last contact or end of the study. The statistical software package SPSS (version 27) was used for statistical analyses. All frequencies were rounded to percentage values. For comparing numbers of deviations in different groups we used the Mann–Whitney U test. All *p* values are two-sided and are referred to as significant at *p* < 0.05. Due to the exploratory nature of this analysis, we did not correct the *p* values for multiple comparisons.

We also conducted multivariate analyses to adjust for possible confounders. Wilcoxon matched-pairs signed-rank tests were performed to detect relevant changes (compared to baseline) in the plasma level of biomarkers, and logistic regressions were used to correlate the concentration of biomarkers with survival and toxicity.

## 3. Results

### 3.1. Patient Characteristics

Between August 2011 and February 2012, 67 patients were registered, of which 56 patients were included in the final analysis. The median follow-up for patients was 22 months. Patients were treated for lung cancer (*n* = 41), esophageal cancer (*n* = 13) or other thoracic malignancies (thymoma, lung metastasis, *n* = 1), either with conventionally fractionated (*n* = 43) or hypofractionated (*n* = 13) radiotherapy. Eight patients were treated with adjuvant radiotherapy after R0 resection, 35 were treated with concurrent chemoradiotherapy, six patients were treated with stereotactic body radiotherapy and seven patients in palliative intent. The median dose was 54 Gy (range: 30–76 Gy). Patient and treatment-related characteristics are shown in Table 1.

### 3.2. Longitudinal Assessment of Blood Biomarkers

**Figure 1:**
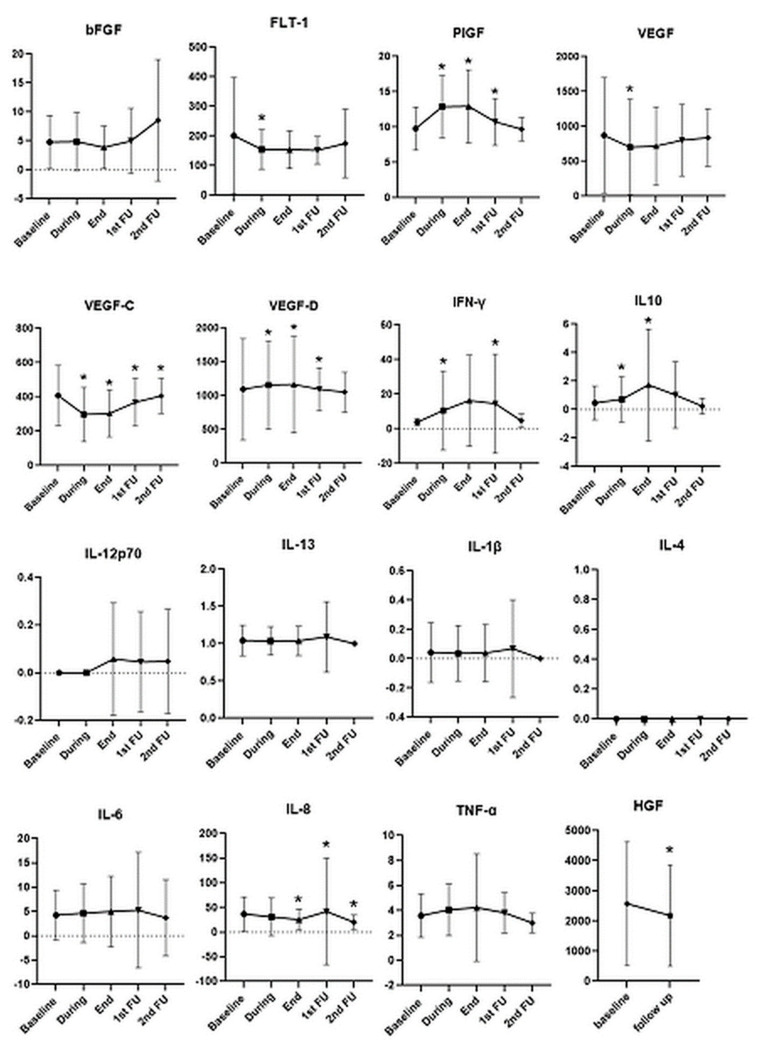
Longitudinal assessment of blood biomarkers in all patients. Detection limits are reported in Table S1. Concentrations are in pg/mL. Statistical differences over time (*p* < 0.05) compared to baseline are marked with asterisks (*). *p* values from Wilcoxon test.

Longitudinal assessment of blood biomarkers showed significant changes in angiogenic and inflammatory biomarkers during and after radiotherapy compared to baseline, (Figure 1). We found an increase in the levels of circulating IL-10 (RTduring, *p* = 0.03; Rtend, *p* = 0.03), IFN-γ (Rtduring, *p* = 0.04; FU1, *p* = 0.002), PlGF (Rtduring, *p* < 0.0001; Rtend, *p* < 0.0001; FU1, *p* = 0.04) and VEGF-D (RT during, *p* = 0.02; Rtend, *p* = 0.04; FU1, *p* < 0.0001) and a significant decrease in those of s-FLT (Rtduring, *p* = 0.045), IL-8 (Rtend, *p* = 0.03; FU1, *p* = 0.01; FU2, *p* = 0.02), VEGF (Rtduring, *p* = 0.007) and VEGF-C during (Rtduring, *p* < 0.0001), at the end of radiotherapy (RTend, *p* < 0.0001) and at follow-up (FU1, *p* = 0.02; FU2, *p* = 0.03). Finally, HGF decreased at follow-up (*p* = 0.013), and bFGF levels were increased at FU2. In contrast, the levels of bFGF, IL-1β, IL-4, IL-12p70 and IL-13 did not show significant variations during treatment and at follow-up.

#### 3.2.1. Association of Biomarkers with Tumor Histology

**Figure 2:**
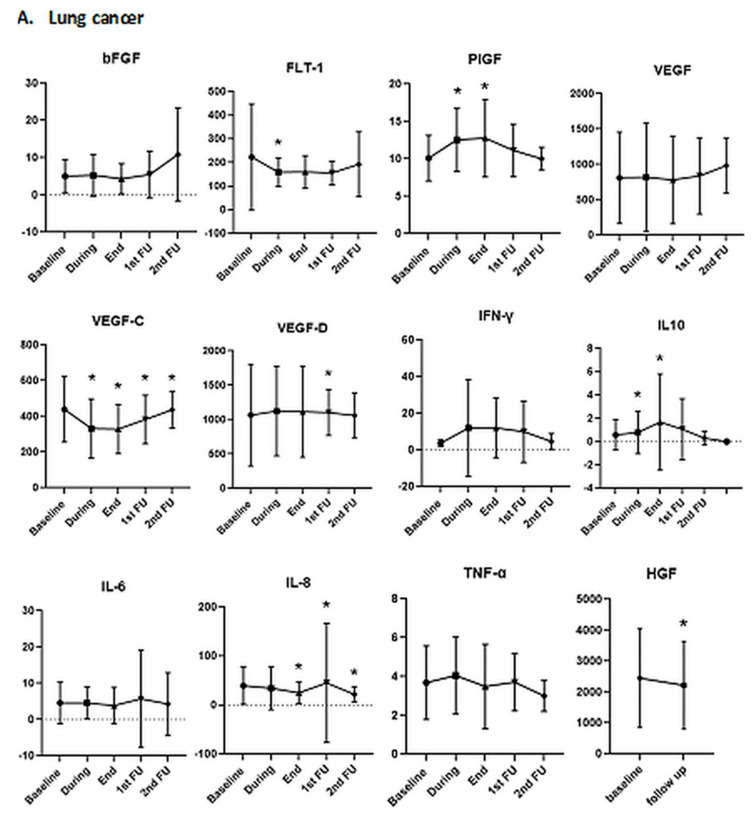

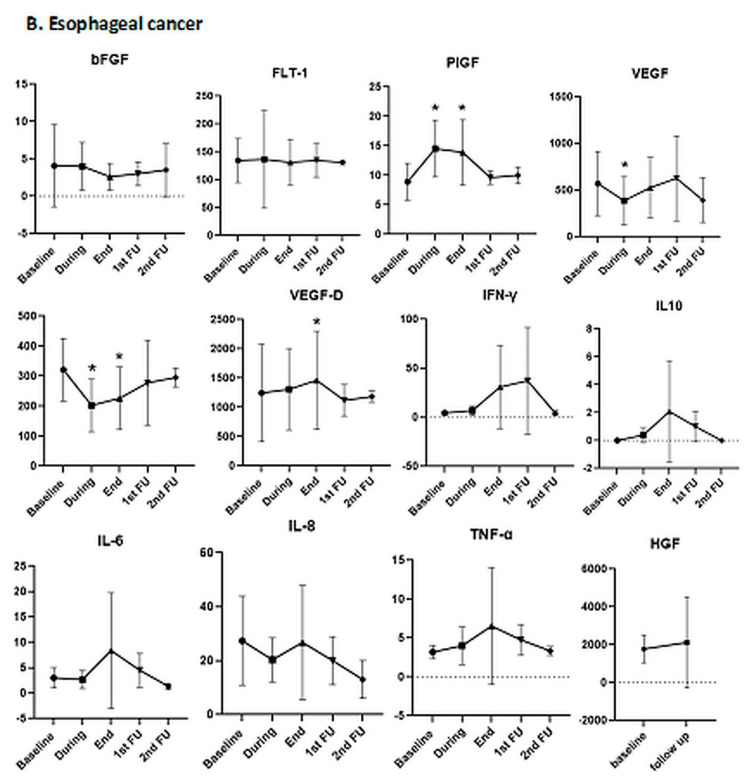
Longitudinal assessment of blood biomarkers (**A**) in lung cancer patients and (**B**) in esophageal cancer patients. Detection limits are reported in Table S1. Concentrations are in pg/mL. Statistically significant changes (*p* < 0.05) compared to baseline are marked with an asterisk (*). *p* values from Wilcoxon test.

We did not detect any difference between biomarker levels at baseline or at any other time point between squamous cell carcinomas and adenocarcinomas, except for the bFGF level at the RTduring time point (*p* = 0.044). Additionally, there was a significant difference at the RTduring time point in the concentrations of sFLT-1 (*p* = 0.03) and VEGF-C (*p* = 0.005) and VEGF-C at RTend (*p* = 0.04) between esophageal and lung cancer patients, but not in any other chemokines. The longitudinal assessment of blood biomarkers in lung cancer patients and esophageal cancer patients is shown in Figure 2. In the subgroup of lung cancer patients, we observed the following differences that were significant or showed a strong trend when compared to baseline: sFLT (at RTduring, *p* = 0.05), PlGF (at RTduring and RTend, *p* = 0.01), VEGF-C (at RTduring and RTend, *p* < 0.001; at FU1, *p* = 0.04), VEGF-D (FU1, *p* < 0.001), INF-γ (FU1, *p* = 0.008), IL-8 (at RTend, *p* = 0.02; at FU1, *p* = 0.03; and at FU2, *p* = 0.03) and HGF (at FU1, *p* = 0.01). Concerning the subpopulation of esophageal cancer only PlGF (at RTduring, *p* = 0.003; and at RTend, *p* = 0.01), VEGF (at RTduring, *p* = 0.02), VEGF-C (at RTduring *p* = 0.001; and at RTend, *p* = 0.03) and VEGF-D (at FU1, *p* = 0.04) showed statistical differences compared to baseline (Figure 2).

#### 3.2.2. Association of Blood Biomarkers with Treatment

**Figure 3:**
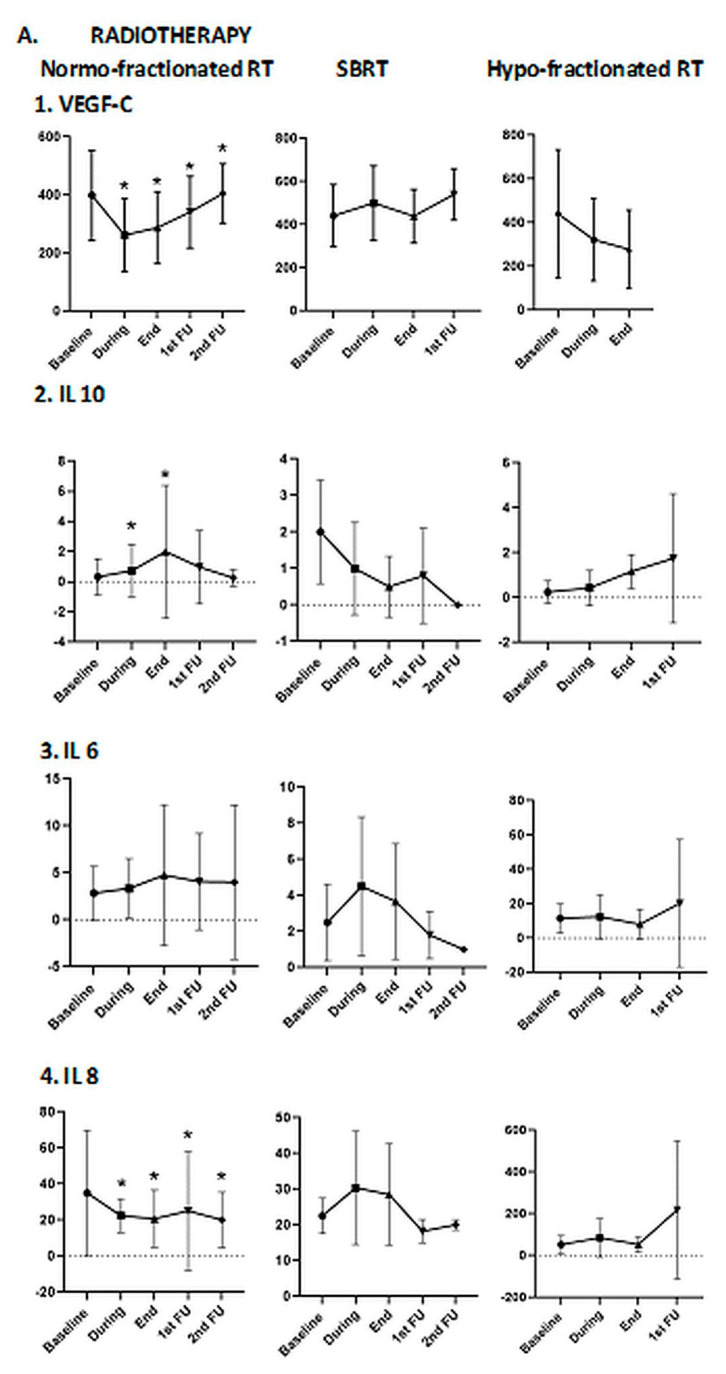

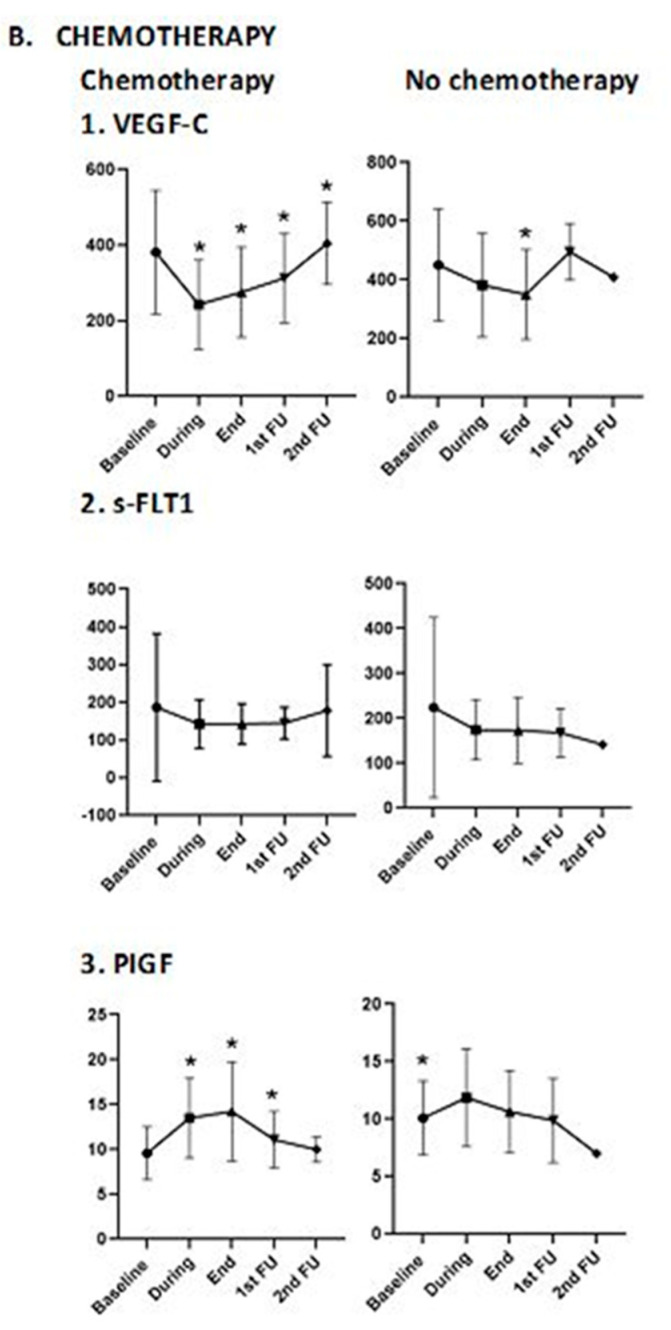
Difference between blood biomarkers with different treatment types: (**A**) radiotherapy (normofractionated vs. stereotactic body radiotherapy vs. hypofractionated radiotherapy); (**B**) chemotherapy. * *p* < 0.05; *p* values from Wilcoxon test compared to baseline.

When stratifying by the type of radiation treatment modalities (normofractionated, hypofractionated and SBRT), there was a difference in the concentrations of IL-6 and IL-10 at the RTbaseline time point (*p* = 0.049 and *p* = 0.018), VEGF-C, IL-6 and IL-8 at the RTduring time point (*p* = 0.019, *p* = 0.041 and *p* = 0.013), IL-8 at the RTend time point (*p* = 0.021) and VEGF-C at the FU1 time point (*p* = 0.020). Notably, IL-6 appeared to be increased during therapy, but changes appeared to be different at subsequent time points depending on modality. When evaluating the impact of chemotherapy on the concentration of the biomarkers at the different time points, the only significant differences were in the levels of sFLT1 and VEGF-C at RTduring (*p* = 0.021 and *p* = 0.005), P1GF at RTend (*p* = 0.017) and VEGF-C at FU1 (*p* = 0.001) between the patients receiving chemotherapy and those who did not. For these cytokines, we performed additional subgroup analyses revealing statistical differences compared to baseline in the group of normofractionated radiotherapy in VEGF-C, IL-10 and IL-8 (Figure 3). Significant differences were detected also in the subgroup of patients in terms of chemotherapy in VEGF-C, s-FLT1 and PlGF (Figure 3).

#### 3.2.3. Correlation between Blood Biomarkers and Survival

Only the levels of three proinflammatory biomarkers were significantly associated with OS. TNF-α at the RTbaseline (HR: 1.360, 95% CI: 1.011–1.829, *p* = 0.017) and FU1 (HR: 1.337, 95% CI: 1.061–1.686, *p* = 0.017) time points were inversely correlated with OS in the whole study population but also in the subgroup of lung cancer patients (RTbaseline: HR: 1.397, 95% CI: 1.009–1.935, *p* = 0.044).

The concentration of IL-8 at the RTduring (HR: 1.014, 95% CI: 1.002–1.026, *p* = 0.017), RTend (HR: 1.015, 95% CI: 1.003–1.028, *p* = 0.016) and FU1 (HR: 1.007, 95% CI: 1.002–1.011, *p* = 0.004) time points was also inversely correlated with OS. This association was confirmed in the subgroup of lung cancer patients for IL-8 level at the RTduring (HR: 1.014, 95% CI: 1.001–1.027, *p* = 0.038), RTend (HR: 1.018, 95% CI: 1.003–1.018, *p* = 0.017) and FU1 (HR: 1.007, 95% CI: 1.002–1.012, *p* = 0.008) time points.

The concentration of IL-6 at the RTend (HR: 1.041, 95% CI: 1.002–1.082, *p* = 0.04), FU1 (HR: 1.139, 95% CI: 1.056–1.228, *p* = 0.001) and FU2 (HR: 1.101, 95% CI: 1.018–1.192, *p* = 0.017) time points was inversely correlated with OS. In the subgroup of lung cancer patients, IL-6 level at FU1 and FU2 correlated with OS (FU1: HR: 1.127, 95% CI: 1.036–1.226, *p* = 0.006, FU2: HR: 1.094, 95% CI: 1.011–1.184, *p* = 0.027).

None of the angiogenesis biomarkers measured correlated with OS. Furthermore, there was no correlation between the changes in the biomarkers during treatment with OS except for IL-6 level, where an increase at the FU1 time point compared to baseline correlated with worse OS (*p* = 0.034).

#### 3.2.4. Correlation between Blood Biomarkers and Toxicity

Seventeen patients (30%) developed radiologic signs of radiation-induced lung disease (RILT) Grade ≥ 1, but only two of them (3.6%) developed clinical symptoms (Grade 2). We did not find any association between the different serial blood biomarkers and a higher incidence of RILT.

## 4. Discussion

We report here changes in a panel of circulating biomarkers of angiogenesis and inflammation. We found an upregulation of the anti-inflammatory cytokine IL-10, which is a potent activator of B lymphocytes during and at the end of radiotherapy, but this change did not correlate with OS. Additionally, there was an increase in the circulating levels of IFN-γ, which is a marker of CTL activation. Paracrine interactions between tumor cells and cancer-associated fibroblasts promote the release of TNF-α, which induces tumor-cell apoptosis, activates the endothelium and granulocytes [22,23] regulating the immune cells and remodels the microenvironment and promote invasion and metastasis [11,24]. In previous studies, elevated circulating levels of TNF-α were associated with advanced/metastatic NSCLC, tumor progression and poor survival [25,26], and our study data are consistent with this association. Additionally, including IL-1β and IL-6 [27] and TNF-α [28] have been linked also with the pathogenesis of esophageal cancer [29].

In our study, circulating levels of TNF-α were increased during radiotherapy and correlated with shorter survival. In addition to TNF-α, two other proinflammatory biomarkers (IL-6 and IL-8) correlated with OS. The increased levels of IL-6 correlated with a worse OS. These results are in line with previous findings by Ryan et al. in stage I lung cancer patients [30], showing that IL-6 and IL-10 were elevated during radiotherapy and downregulated at the end of treatment. IL-6 enhances T-cell and B-cell function; inhibition of IL-6 reduces lymphoproliferation [22,31] and stimulates the growth and differentiation of B-cells and T cells [26]. Prior studies have reported that serum cytokine levels correlated with survival in lung cancer patients [11,32]; in particular, IL-6, IL-8 and TNF-α. IL-6 and IL-8 significantly correlated with surgical treatment outcomes in stage I NSCLC patients, and the combination of IL-6 and IL-8 increased the prediction value. Dysregulated expression of cytokines in the IL-6 family and downstream receptor signaling are frequent events in cancer and are often associated with poor clinical outcomes [33,34,35,36,37]. In this regard, the protumorigenic effects of IL-6 cytokine family members are elicited by both direct intrinsic effects on cancer cell activities (for example, cell proliferation, survival, migration, invasion and metastasis) and indirect effects on the stromal cell compartment, such as modulation of inflammation, immunosuppression and angiogenesis, which shape the interaction with the tumor microenvironment [33,34,35,36,37,38]. As reported by Wang and Yang [39], serum levels of IL-6 after treatment can be used as an indicator to understand which patients might need more aggressive therapy approaches. They reported a significant reduction in the IL-6 serum level in radiotherapy responders compared to nonresponders. In another study, higher baseline serum and bronchoalveolar lavage (BAL) fluid IL-8 and serum VEGF levels were associated with shorter survival, showing that lung cancer is associated with upregulation of IL-6 and IL-8. In our study, patients with lung cancer showed a drop in IL-6 levels during and at the end of treatment, while patients with esophageal cancer showed a decrease in IL-6 levels during treatment but an increase at the end of treatment. In several studies for patients undergoing ICB, a decrease in IL-6 levels was associated with improved PFS or OS [40,41]. These findings suggest the possibility that patients who do not respond under ICB might benefit from additional radiotherapy, which in lung cancer patients might be performed during or at the end of treatment, while in esophageal cancer patients, this combination might be beneficial during treatment. In our study, this difference might also be due to the small number of patients with esophageal cancer enrolled and thus should be interpreted with caution.

Serum IL-8, along with IL-6, was reported as significantly decreased in radiotherapy responder breast cancer patients when compared to baseline levels [39]. In contrast, De Sanctis et al. reported no variations in IL-8 serum level at 4 weeks after radiotherapy when compared to baseline [42], whereas Muraro et al. reported a lower IL-8 level in breast cancer patients compared to controls at baseline and a significant increase 1 month after SBRT [43]. Several studies reported increases in IL-8 levels in patients with various cancers, and higher circulating IL-8 levels seem to correlate with a more advanced stage, higher grade and greater tumor burden [44,45,46]. Pilot data from small retrospective cohorts have also recently suggested that increases in serum IL-8 during treatment may be predictive of resistance to ICBs. Schalper et al. found that higher pretreatment serum IL-8 correlated with lower survival across tumor types [47]. The prognostic value appeared to be consistent across patients who were treated with either of two types of ICBs (i.e., inhibitors of PD-1 or CTLA-4) as single agents or in combination, with an mTOR inhibitor (everolimus) and with chemotherapy (docetaxel). These results suggest that greater IL-8 expression in the tumor correlates with higher circulating serum IL-8, an immunosuppressive myeloid-enriched tumor microenvironment with decreased T-cell responsiveness and poor prognosis in multiple tumor types (independently of the type of systemic therapy) [46,47]. According to Yuen et al. and Schalper et al., high systemic and tumor-associated IL-8 levels that correlate with the reduced benefit of anti-PD-L1 therapies and can reverse the impacts of IL-8-mediated myeloid inflammation will be essential for improving outcomes of patients treated with immune checkpoint inhibitors [47,48]. Collectively, these data indicate that radiotherapy might be beneficial by decreasing IL-8 levels during and at the end of treatment for lung cancer patients and for esophageal cancer patients during treatment, suggesting that ICBs should be administered early in the treatment or after the end of treatment but not later, as also suggested by data from the PACIFIC trial [16].

Additionally, in the subgroup of patients treated with SBRT, both IL-6 and IL-8 were increased (Figure 3), suggesting that the effect of radiotherapy on tumor immunogenicity might be dependent on the dose per fraction, but due to the small sample size, these results are not easy to interpret. These data are consistent with the report by Vanpouille-Box et al., who demonstrated the induction of DNA exonuclease Trex1 by radiation doses above 12–18 Gy in different cancer cells attenuated their immunogenicity by degrading DNA that accumulates in the cytosol upon radiation. [49]. Cytosolic DNA stimulated secretion of IFN-β by cancer cells following activation of the DNA sensor cGAS and its downstream effector STING [49]. Repeated irradiation at doses that do not induce Trex1 amplifies IFN-β production, resulting in the recruitment and activation of Batf3-dependent dendritic cells. This effect is essential for the priming of CD8^+^ T-cells that mediate systemic tumor rejection (outside the irradiation field or “abscopal effect”) in the context of ICB.

In the past several years, attempts have been made to evaluate the role of several cytokines and chemokines in the early prognostication of RILT [11,18,50,51,52,53,54,55,56], as well as in the development of radiation-induced liver disease [57,58]. In RILT, changes in circulating IL-6 and IL-10 levels early during radiotherapy were reported to be significantly associated with a higher incidence of RILT in multivariate analysis (*p* = 0.011) [11,59,60]. IL-6 levels before, during and after thoracic radiotherapy were reported to be significantly higher in those who developed pneumonitis [59]. A low baseline level of IL-8 expression was reported to be highly associated with RILT in two independent studies. In our study, we could not identify a significant association, likely due to the low incidence of RILT in our study.

Our study has several limitations. As the study was an expansion of a prior study designed to evaluate the role of CCL18 in predicting radiation-induced lung disease [18] after radiotherapy, which included a heterogeneous group of thoracic malignancies. However, these data suggest that there is a global response to thoracic irradiation reflected by an increase in cytokine levels and a specific response of the tumor microenvironment to treatment. Another limitation is the relatively small number of patients included in the study. However, while this exploratory analysis might be underpowered and can be only regarded as hypothesis-generating, especially regarding the correlations with the OS, it is important to note that our results concerning the longitudinal changes of blood biomarkers are in line with previous reports in stage I–III lung cancer and esophageal cancer [30,61,62]. Larger studies are warranted to validate these results. These limitations notwithstanding, our study addresses the unmet need for a biomarker (or biomarkers) to guide the implementation of radiotherapy in combination with ICB, which is yet to be achieved clinically.

## 5. Conclusions

In summary, our study identified changes in circulating TNF-α, IL-6 and IL-8 levels as potential biomarkers of the systemic immunomodulatory effects of radiotherapy. Noninvasive blood biomarkers might help stratify patients that could benefit from immunotherapy and help with the optimal scheduling of combinations of radiotherapy with ICBs. These hypothesis-generating results warrant further investigation of these circulating biomarker candidates in more homogeneous and larger patient populations to better stratify patients eligible for combined ICB and radiotherapy-based treatments.

## Figures and Tables

**Table 1:** Patient characteristics.

Variable	Number of Patients (%)
Gender	
Male	38 (68%)
Female	18 (32%)
COPD	
COPD Gold 3–4 *	35 (62.5%)
COPD Gold 0–2	8 (14.3%)
Tumor type	
Lung cancer	41 (73.3%)
Esophageal cancer	13 (23.1%)
other	2 (3.6%)
Histology	
Squamous cell carcinoma	24 (42.9%)
Adenocarcinoma	20 (37.5%)
Small cell lung cancer	3 (5.4%)
Large cell carcinoma	2 (3.6%)
other	7 (12.5%)
Tumor stage	
T1	8 (14.3%)
T2	16 (28.6%)
T3	22 (39.3%)
T4	10 (17.9%)
N0	17 (30.4%)
N1	7 (12.5%)
N2	23 (41.1%)
N3	8 (14.3%)
M0	49 (87.5%)
M1	7 (12.5%)
Type of treatment	
Adjuvant	8 (14.3%)
Concurrent chemoradiation	35 (62.5%)
Stereotactic body radiotherapy	6 (10.7%)
Palliative radiotherapy	7 (12.5%)
Chemotherapy	
Yes	35 (62.5%)
No	21 (37.5%)

Abbreviations COPD = chronic obstructive pulmonary disease, * COPD was dichotomized as not significant (COPD GOLD 0–2) and significant (COPD GOLD 3–4).

## Data Availability

All data are available and presented in the manuscript.

## Supplementary Materials

The following are available online at https://www.mdpi.com/article/10.3390/cancers13225725/s1, Figure S1: Study design diagram, Table S1: List of all noninvasive biomarkers included in the study.
