# Supplementary material for: Changes in Blood Biomarkers of Angiogenesis and Immune Modulation after Radiation Therapy and Their Association with Outcomes in Thoracic Malignancies
Source: Cancers (Basel). 2021 Nov 16;13(22):5725. doi: 10.3390/cancers13225725 (PMC8616228; doi:10.3390/cancers13225725)
Supplement: Supplementary file 1 [file cancers-13-05725-s001.zip › Supplementary Table S1.pdf]

Table S1. List of all non-invasive biomarkers included in the study.

	Marker	Detection limits (pg/ml)
**Angiogenesis**		
Vascular endothelial growth factor	VEGF	1.23-3956.56
Vascular endothelial growth factor C	VEGF-C	58.64-51293.96
Vascular endothelial growth factor D	VEGF-D	16.05-46970.61
Placental growth factor	PlGF	0.69-2455.22
Basic fibroblast growth factor	bFGF	1.01-3886.26
Soluble fms-like tyrosine kinase 1	sFLT1 (sVEGFR1)	4.41-16419.24
**Inflammation**		
Interleukin 10	IL-10	0.19-706.40
Interferon-γ	IFN-γ	0.80-2758.93
Interleukin-12p70	IL-12p70	0.28-997.30
Interleukin-13	IL-13	1.41-973.81
Interleukin-1β	IL-1β	0.32-1127.14
Interleukin-4	IL-4	0.13-494.91
Interleukin-6	IL-6	0.40-1451.13
Interleukin-8	IL-8	0.29-1108.53
Tumor necrosis factor-α	TNF-α	0.20-739.12
**Growth factor/invasion**		
Hepatocyte growth factor	HGF	156-10,000
