# Supplementary figures and images for: Changes in Blood Biomarkers of Angiogenesis and Immune Modulation after Radiation Therapy and Their Association with Outcomes in Thoracic Malignancies
Source: Cancers (Basel). 2021 Nov 16;13(22):5725. doi: 10.3390/cancers13225725 (PMC8616228; doi:10.3390/cancers13225725)

**Supplementary Figure S1.** Study design diagram

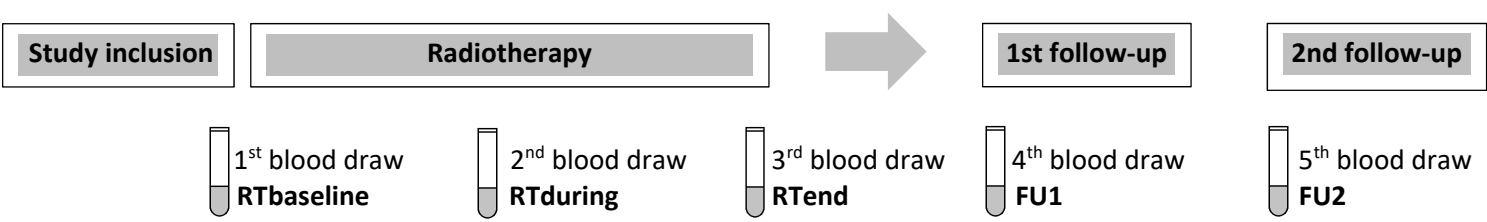

Supplement: Supplementary file 1 [file cancers-13-05725-s001.zip › Figure S1.pdf]
